# Erie County Medical Society

**Published:** 1884-07

**Authors:** Edward Clark

**Affiliations:** Secretary


### Erie County Medical Society.

The semi-annual meeting of the Erie County Medical Society was held at the Buffalo Medical College, June, 10, 1884.

The meeting was called to order by the President, Dr. J. C. Greene.

The following members were present: Drs. F. H. Potter, Daggett, Daniels, Fell, Walsh, Hubbell, Cronyn, Greene, Gay, Lothrop, Marcley, Ingraham, Lynde, Hopkins, Howe, Storck, Phelps, Harrington, Mann, Wyckoff, Warren, Coakley, Barker, Cary, E. E. Storck, B. G. Long, Abbott, Pryor, Diehl, Hill, E. H. Long, Park, Gregory, Van Peyma, Dorland, Stockton, Krug, C. A. Ring, Hoyer, Schada, Benj. Lothrop, Frederick Davidson, W. W. Potter, Crawford, Witthaus, Hebenstreib, Mynter, Folwell, Niemand, Hauenstein, Frank, Nichell, Bartow, Wetzel, McBeth, Brecht, Dambach, Mickle, Wall, Montgomery, Samo, Keene, Boies, McPherson, Bartlett, Hawkins, J. H. Potter, Bauer, Persons, Root, Ballou, Wm. Ring, Clark.

The minutes of the annual meeting, and the minutes of the special meeting of April 8th, were read.

Before the minutes were adopted, Dr. Hopkins said he wished to call the attention of the society to Section 16, Article 4 of the By-Laws, which provides that "on a division, the names of all members voting for, or against, a question or motion, shall be entered upon the minutes." According to the reading of the minutes, this rule had not been observed.

Dr. Wyckoff moved that the Secretary be instructed to correct the minutes so that the ayes and noes appear in the report of the society's meeting.

Dr. Hopkins moved that inasmuch as the minutes were incorrectly reported, that portion of the minutes relating to the admission of Drs. Tremaine and Heath be corrected, so as to read as follows: "That inasmuch as Drs. Tremaine and Heath have, at a previous meeting of this society, been invited to participate in the proceedings, the rules be suspended, so as to construe such invitation to include the privilege of voting upon any question before the society at this meeting or any meeting hereafter."

During the discussion on the resolution of Dr. Hopkins, Drs. Tremaine and Heath said that the society need not discuss any further the question of their membership, for they did not wish to be members of the society, and while they were thankful for past courtesies extended to them, they would now withdraw.

After one or two minor corrections, the motions offered by Drs. Hopkins and Wyckoff were adopted, and the minutes, as corrected, were approved.

Dr. Dorland, chairman of the Committee on Membership, reported as follows: "The following gentlemen presented their credentials at the January meeting, in 1884. The committee find that the diploma of each was granted by a medical college in good and regular standing, and we recommend the following named gentlemen for membership, viz.: Herman Bauer, Roswell Park, R. M. Root, F. R. Campbell, Julius H. Potter, Andrew F. Helwig, Wm. B. Hawkins, Alpheus Prince, Herbert Mickle, Stephen Y. Howell, Louis Carmer.

Report received and recommendation adopted.

Dr. Barker moved that the new members be introduced to the society by the chairman of the Committee on Membership. Carried.

The new members were introduced by Dr. Dorland.

Applications for membership were received from the following gentlemen and referred to the Committee on Membership, viz.: Wm. Pask, James Porter, H. Parmenter, A. B. Wilson, Wm. G. Ring, F. W. Hinkle, F. P. Vanderberg.

The Board of Censors, through their chairman, Dr. Storck, submitted the following report:

Your Board of Censors would respectfully report that inasmuch as the minutes of the society give a full account of the doings of the censors from the time of the annual meeting in January down to the special meeting of April 8th, it is deemed necessary to make a report, at this time, only of work accomplished subsequent to the date last mentioned. In obedience to the resolutions of the society condemning the bills establishing certain medical colleges in this city, at that time pending before the Legislature, the censors at once opened correspondence with the authorities at Albany, and learned that the objectionable bill relating to the Seminary of our Lady of Angels known here as Niagara University, had already passed the Senate, having passed the Assembly before knowledge of its existence came to the censors, and was already in the hands of the Governor.

At the request of the censors, a hearing before the executive was set for the 12th of April. The advocates of the bill were represented at this hearing by several members of the medical faculty, and by the trustees of the Seminary of our Lady of Angels and by their attorney, Mr. Morey, while the society was represented by a member of the Board of Censors and their counsel, Mr. J. F. Milburn. This gentleman ably presented the objections recited in the resolutions condemning the bill, adopted at the special meeting of April 8th, and the arguments and influence of the other side seemed to be insufficient to break the force of their effect.

As the result of the hearing, the Governor did not sign the bill, and it therefore did not become a law.

Subsequently a new bill was introduced. This was brought to the notice of the censors, and found to be free from the faults of its predecessor of 1883, and of March 11, 1884. This passed the Legislature and was signed by the Governor.

This last bill provides:

"SECTION 6. Whenever, in the opinion of the regents of the university, the state of literature in the said seminary and the value of property, according to the regulations of the said regents, shall justify the same, said regents may, on the petition of the trustees, by an instrument under their common seal, erect the said seminary into a college, with such name and such number of trustees, and on such conditions, and with such powers and privileges, and subject to such regulations and restrictions, conformable to law, as the said regents may deem proper, and the said college shall have the right to maintain a medical department, and any other department of learning that is maintained by any college or university in this State, and may maintain any department thereof, and enjoy and exercise all or any of the powers, rights, and franchises thereof in the county of Erie, and the trustees of said college shall have the power and right to grant and confer any degree which may be or is granted and conferred by any college, university or other institution of learning in this State, legally authorized so to do, and every diploma given in testimony thereof shall entitle the possessor to all the immunities which, by the law or usage, are allowed to the possessors of similar diplomas granted by any college, university or other institution of learning in this State, and the trustees of such college may grant and confer the degree of Doctor of Medicine, but only upon the recommendation of the board of medical professors of said college, and of at least three curators of the medical profession fully and legally qualified to practice medicine and surgery, to be appointed by said trustees, and who shall not in any manner be connected with said college. But no person shall receive a diploma conferring such degree unless he be of good moral character and of the age of twenty-one years, and unless he shall have received a good English education, and shall have pursued the study of medicine and the sciences connected therewith at least three years, after the age of sixteen years, and shall have received instruction from some physician and surgeon legally qualified to practice his profession until he is qualified to enter a medical college, and also shall have attended at least three complete courses of lectures in the medical department of some legally incorporated university or medical school, the last of which courses shall have been so attended in the medical department of said college."

In comparing the bills, it will be observed that the changes introduced in this bill are directly in the line of the criticism made upon the previous attempts at legislation by this college, as expressed in the resolutions adopted at the special meeting of April 8th.

In the case of the bill to establish the so-called "Mohawk College," equally important amendments were secured, so that the bill specially states that it does not legalize the said college, but only legalizes the diplomas thereof granted to its students. In this amended form the bill has passed the Legislature, and is reported as having been signed by the Governor.

In the prosecution of this work the Board of Censors has incurred an expense of $75.00, the items being $25.00 for the opinion of Rogers, Locke & Milburn upon the competency of the charter of the Niagara University, and $50.00 for the services of counsel at the hearing before the Governor, April 15th.

Your board would further report that conclusive evidence is in the hands of the censors against a number of persons still practicing medicine in this city and county in violation of the laws of the State, notwithstanding notice having been served upon them to desist from so doing. The chairman of the board has been directed to prosecute them forthwith. Warrants have been issued by the Police Court, and were served, on Monday last, upon the following named persons, viz.:

Frederick Dellenbaugh, 173 Broadway.

Caspar Retel, 259 Broadway.

William Volker, 333 Ellicott street.

Mrs. N. Kuhn, 290 Ellicott street.

Mrs. J. H. Matteson, 248 North Division street.

The board would ask for an appropriation of sufficient funds to proceed with the prosecution.

EDWARD STORCK,
H. R. HOPKINS,
P. W. VAN PEYMA.

After the report of the Board of Censors was read, Dr. Hopkins moved that it be received and placed on file, which was carried.

Dr. Hopkins, from the Committee on Legislation, presented the following report, which was received and placed on file:

*To the Medical Society of the County of Erie:*

Mr. President and Gentlemen—Your Committee on Legislation would report: One year has passed since your committee was appointed, and upon reviewing the events of the year and the progress which has been made in the particular work in which it has been engaged, there is found abundant reason why the society should congratulate itself upon the success of its efforts and upon the general state of the movement for improved methods in the manner of licensing physicians. This time last year, the question of a State Examining Board was one securing but slight attention from physicians, and none at all from the public at large; but for the last six months there has been no medical topic of equal prominence with this in professional circles, and the files of our daily papers show repeated instances of the interest extending beyond the profession, and that the public at large are awakening to an earnest appreciation of its importance. At the time of the last stated meeting of this society, in January of this year, many of our more sanguine friends were positive that our efforts were to be crowned with entire and speedy success by the passage of the State Faculty Bill by the Legislature then sitting, and your committee is of the opinion that such would have been the case, but for the action of the State Medical Society in declining to recommend the bill which its own Committee on Legislation had adopted and was vigorously endeavoring to pass. This action was as unexpected as it was calamitous, and furnishes one more illustration of the power of a well-organized and alert minority, more particularly when that minority represents a long-established and lucrative monopoly. Such monopolies relinquish their power only after a long and desperate struggle, the will of the majority being evaded or defied as the result of the temporary triumph of the well-organized few over the unorganized many. There was in this failure, however, no cause for disheartenment, and much that should encourage. The most that its enemies can claim is that they defeated the particular bill then before the Legislature, and this was done by appealing to the well-known and characteristically conservative disposition of our profession, with the claim that more time should be taken to consider this question; that the matter was one of great importance and should not be jeopardized by too hasty action, and that it would do no harm to lay the matter over, for more consideration, another year, etc. Under this skillfully developed, specious and disingenuous special pleading the State society voted to lay the matter over. Emboldened by this partial victory the enemies of State license moved to kill the main issue by referring it to a committee specially prepared for this purpose, and in this they were most emphatically checked and beaten. The society then, by a fair majority, voted to endorse the main principle of this matter of State license, and referred the subject to a special committee, with instructions to report a bill for the consideration of the society at its next meeting.

Your Committee on Legislation is represented on this special committee, and is informed that the work is to be vigorously pushed upon the line essentially introduced to the profession in the Erie county bill. Our society, therefore, can claim, as the result of the first year's effort in the cause of securing State license for physicians, that the question has been established as the leading topic for discussion all over the State; that the plan proposed by this society has been discussed, considered and approved by the county societies of Albany, Allegheny, Broome, Cayuga, Chautauqua, Chemung, Fulton, Genesee, Herkimer, Lewis, Livingston, Monroe, Niagara, Oneida, Onondaga, Oswego, Rockland, Schenectady, Steuben, Tioga, Tompkins, Wayne, Westchester, Wyoming and Yates; that the ground plan of the measure has been approved by the societies of New York and King's counties, and favorably considered by Chenango, Jefferson, Ontario, Orleans, Saratoga, Schuyler, Seneca, Warren, and nearly all the others. That the topic was by all means the most prominent one at the late meeting of the State society, inasmuch as its discussion took up nearly all the second day of the session, and is to be kept before the profession during the present year, in the hope that a plan may be devised which will command the support of the profession at large, as the best means to correct the fault in our present mode of licensing physicians, a fault which even the enemies of the movement are forced to admit.

In conclusion, the committee would recommend that the society continue its interest in this work. Great progress has been made, but the enemies of the cause are still determined, active and powerful. The initiation being taken by the medical society of the State does not prevent this society from finding and doing telling work.

The interest shown by the various county societies throughout the State in our efforts, would indicate that this society has the opportunity to make for itself upon this question a position of no inconsiderable influence and power, and it is, therefore, recommended that the Medical Society of the County of Erie hereby reaffirms its sense of the importance of the effort to secure an improved method in the licensing of physicians, and believes that this improvement will only be reached when a State board shall absolutely separate the licensing and teaching power. In the furtherance of this end it is recommended that there shall continue to be a Committee on Legislation.

Respectfully submitted,

H. GREENE, *Chairman*,
EDWARD STORCK,
M. D. MANN,
F. S. CREGO,
A. R. DAVIDSON,
A. H. BRIGGS,
H. R. HOPKINS,
*Committee on Legislation.*

Under the head of "Miscellaneous Business," Dr. Storck offered the following resolution, which was adopted:

*Resolved*, That the report of the Board of Censors be adopted, and the Treasurer of this society be authorized to pay, on the order of the President, to the chairman of the Board of Censors, seventy-five dollars, the amount incurred for expenses in its work, as reported.

Under the same head Dr. Barker offered the following proposed amendments to the By-Laws, viz.:

(1.) To amend Sec. I of Art. 5 so that it shall read: "Every physician or surgeon residing in the county of Erie who may hereafter wish to become a member of this society shall make his application to the Secretary, who shall present it, at a regular meeting, to the society. It shall be referred to the committee on membership, who shall report the result of their investigation, accompanied with a recommendation for or against the applicant, at the next regular meeting of the society. The applicant shall be admitted by a majority vote of the members present."

(2.) To amend Sec. IV of Art. 5 by striking out the words, "pay the Treasurer one dollar," so that the section would read, "Every member, when admitted, shall sign the By-Laws, and be entitled to a certificate of membership."

Under the same head Dr. Gay moved that, inasmuch as the rooms now used by the society are too small to accommodate the large membership, a committee of three be appointed by the President to procure information in regard to rooms for the society's use further down town than the rooms now used by the society, such committee to report at the next regular meeting.

Carried.

The President appointed, as such committee, Dr. C. C. F. Gay, chairman, Dr. U. C. Lynde and Dr. H. R. Hopkins.

Dr. Gay also moved that the Board of Censors be empowered to investigate and inquire into the legality of two institutions in this city, viz.: Dr. Don's dispensary and Mrs. Kimball's maternity home.

Carried.

Dr. Ring suggested that the committee on rooms confer with the Commissioners of the City and County Hall, and see if rooms in that building could be secured for the society's use.

Under the head of "Communications from Abroad," the following communication from the special committee on the State Examining Board of the State Society was received and placed on file:

New York, May 31, 1884.

*To the President of the Medical Society of the County of Erie:*

Sir—At the annual meeting of the Medical Society of the State of New York, held in February, 1884, a special committee was appointed for the purpose of preparing a bill relative to the establishment of a State Examining Board, said bill to be presented for the consideration of the State Medical Society in February, 1885.

At a recent meeting of the committee it was resolved to solicit the views of those chiefly interested. To that end you are requested to bring the subject to the attention of the body over which you preside, and to communicate any action it may take in the matter to the undersigned, prior to October 15, 1884.

Yours respectfully,

H. G. PIFFORD,

*Chairman Special Committee.*

No. 10 W. 35th Street, New York.

After the above communication was read, Dr. Hopkins offered the following resolutions, which were adopted :

*Whereas*, This society has received a communication from the special committee of the Medical Society of the State of New York appointed for the purpose of preparing a bill relative to the establishment of a State Examining Board, in which that committee solicits the views of this society upon the subject they have in hand; therefore—

*Resolved*, That the Medical Society of the County of Erie regards the question of a State Examining Board as one of vital and imminent necessity and importance.

*Resolved*, That it is the belief of this society that such Examining Board should be composed of physicians appointed in the interest of the public, and should be men having no connections liable to conflict with the full and impartial exercise of their duty as examiners.

*Resolved*, That in the event of the adoption of these resolutions, they be officially transmitted, with the vote adopting the same, to the committee on State Examining Board.

There being no further business before the meeting, on motion of Dr. Lothrop, the society adjourned.

EDWARD CLARK, *Secretary.*

